# Cattle grazing results in greater floral resources and pollinators than sheep grazing in low‐diversity grasslands

**DOI:** 10.1002/ece3.8396

**Published:** 2022-01-13

**Authors:** Jasmine Cutter, Torre Hovick, Devan McGranahan, Jason Harmon, Ryan Limb, Jonathan Spiess, Benjamin Geaumont

**Affiliations:** ^1^ School of Natural Resource Sciences—Range Science Program North Dakota State University Fargo North Dakota USA; ^2^ Hettinger Research Extension Center North Dakota State University Hettinger North Dakota USA; ^3^ School of Natural Resource Sciences—Entomology Department North Dakota State University Fargo North Dakota USA

**Keywords:** butterfly, cattle, Conservation Reserve Program, grazing, patch‐burn grazing, pollinator, sheep

## Abstract

1. Land‐use and land‐cover change associated with agriculture is one of the main drivers of biodiversity loss. In heavily modified agricultural landscapes, grazing lands may be the only areas that can provide essential resources for native grassland species. Management decisions, such as choice of livestock species, affect the extent to which grazing lands provide suitable habitat for native species such as pollinators.
2. Our study compared how sheep versus cattle herbivory affected floral resources and butterfly abundance across low‐diversity, former Conservation Reserve Program (CRP) pastures managed with patch‐burn grazing.
3. Across all years (2017–2019), flowering species richness and abundance were significantly higher in cattle pastures than sheep pastures. On average, we recorded 6.9 flowering species/transect in cattle pastures and 3.8 flowering species/transect in sheep pastures. The average floral abundance per transect was 1278 stems/transect in cattle pastures and 116 stems/transect in pastures grazed by sheep.
4. Similarly, we observed higher butterfly species richness, diversity, and abundance in cattle than in sheep pastures. In cattle pastures, we observed an average of 75 butterflies and 6.75 species per transect, compared with an average of 52 butterflies and 3.37 species per transect in sheep pastures. However, the butterfly community composition did not significantly differ between grazing treatments likely because agricultural‐tolerant, habitat generalists comprised the majority of the butterfly community. Five generalist butterflies comprised 92.3% of observations; *Colias philodice* was the most abundant (61% of observations). *Speyeria idalia* and *Danaus plexippus*, two butterflies of conservation concern, comprised less than 0.5% of butterfly observations.
5. Our results, which are among the first attempt quantifying butterfly use of post‐CRP fields grazed by livestock, show that increased precipitation and cattle grazing promoted higher forb abundance and richness. However, additional interventions may be needed to enhance floral resources to sustain and improve pollinator diversity in these landscapes.

## Abstract 2

Sheep grazing resulted in significantly lower floral abundance compared with cattle grazing. However, butterfly communities were fairly homogenous and did not differ between grazers.

## 1 INTRODUCTION

Land‐use and land‐cover change associated with agriculture is one of the main drivers of biodiversity loss (Deguines et al., 2014; Sala et al., 2000; Tscharntke et al., 2005). The conversion of grasslands to crop fields greatly limits the resources available to native species, resulting in biodiversity loss, which threatens the delivery of ecosystem services in agricultural landscapes (Balvanera et al., 2006; Cardinale et al., 2012; Hooper et al., 2005). Maintaining biodiversity and ecosystem services delivery is contingent on the quantity of perennial cover in agroecosystems, as well as the diversity and abundance of resources that remaining semi‐natural areas provide for wildlife species (Duelli & Obrist, 2003; Hendrickx et al., 2007; Ockinger & Smith, 2007). In the United States, perennial cover in agricultural landscapes is often the result of the Conservation Reserve Program (CRP) operated by the U.S. Department of Agriculture, which pays farmers to replant marginal croplands with perennial grasses and legumes (Farm Service Agency, 2019). The amount of biodiversity present on the landscape is greatly affected by how these semi‐natural areas are managed (Benayas et al., 2009), the residual effects of previous land uses (Hahn & Orrock, 2015; Moranz et al., 2012), and the influence of commodity crop prices (Wright & Wimberly, 2013) and policies that incentivize conservation practices (Ravetto Enri et al., 2020) on the profitability of different land uses. There is an urgent need to quantify the effects of management choices on biodiversity in agricultural landscapes.

In heavily modified agricultural landscapes, grazing lands may be the only areas that can provide essential vegetation and structural resources for native grassland species, but their utility for native species depends on how they are managed (Morandin et al., 2007; Polasky et al., 2005). When CRP contracts expire after 10–15 years, landowners can re‐enroll in CRP, transition back to row crops, or use the established perennial cover as forage for livestock. Using former CRP lands for livestock production provides the incentive of livestock income and can benefit wildlife by maintaining perennial vegetation cover (Morandin et al., 2007). However, decisions about stocking rate (Herrero‐Jáuregui & Oesterheld, 2018), grazing regime (Jacobo et al., 2006; Pittarello et al., 2017), grazing duration (Ravetto Enri et al., 2017), grazing season (Hart et al., 1988), and livestock species (Celaya et al., 2010) can influence the structure and composition of the vegetation in grazed landscapes (Albon et al., 2007; Celaya et al., 2010; Rook & Tallowin, 2003). In particular, the influence of livestock species on vegetation and higher trophic levels has often been overlooked when considering grazing management decisions (Rook et al., 2004; Tóth et al., 2018).

Grazer species affects plant community characteristics in direct and indirect ways due to differences in animal physiology and associated differences in diet needs and preferences (Allred et al., 2013; Launchbaugh & Walker, 2006; Rook et al., 2004). Even species that share the same digestive system type (i.e., ruminants), such as sheep (*Ovis aries*) and cattle (*Bos taurus*), can exhibit different selection preferences due to rumen volume and mouth dexterity (Hanley, 1982). Sheep are able to use their mouth and bottom teeth to bite lower on the plant and to continue grazing as plant height decreases (Rook et al., 2004). Additionally, sheep often selectively graze forbs (Dumont et al., 2011) and are able to discriminate between plants at a fine scale (Ginane et al., 2015). Greater forb consumption by sheep can result in a different plant community composition when compared to grasslands grazed by cattle exclusively (Dumont et al., 2011; Ravetto Enri et al., 2017).

In particular, there is evidence that due to their preference for forbs, sheep can have a negative effect on floral abundance and pollinator abundance compared to areas grazed by cattle (Carvell, 2002; Ravetto Enri et al., 2017; Tóth et al., 2018). Patch‐burn grazing is a management strategy that may be able to mitigate the detrimental effects that sheep have on flower abundance (Carvell, 2002; Ravetto Enri et al., 2017; Tóth et al., 2018). Combining fire and grazing can create a “magnet effect” (Archibald et al., 2005), which focuses herbivore grazing in recently burned patches, allowing unburned areas to have reduced grazing pressure and potentially greater opportunity for floral expression (Allred et al., 2011). If sheep focus their grazing on recently burned areas, that may alleviate some of the grazing pressure on flowers in other portions of the management unit. If sheep grazing patterns are less responsive to the burned patch and forbs continue to be disproportionately consumed, then managers trying to promote pollinators and other forb‐dependent species in areas grazed by sheep may need to take additional actions to maximize forb abundance and vegetation structure. Thus, choice of grazer and grazing management can greatly affect plant communities, which can influence species of conservation concern in higher trophic levels.

Pollinators are an ideal indicator group for assessing the impacts that grazing and grazing management have on grassland species. Bees and butterflies are dependent on plant communities for forage, host plants, and nesting resources and thus may be influenced by how grazing shapes vegetation composition and structure (Di Giulio et al., 2001; Fourcade & Ockinger, 2017; Liu et al., 2015; Soderstrom et al., 2001; Tadey, 2015). In particular, butterflies can act as a useful indicator group because they require a variety of vegetation structure and composition throughout their life cycle and adults are mobile and can rapidly react to changes in their environment (Farhat et al., 2014; Fleishman & MacNally, 2003; Kremen et al., 2007; Mac Nally et al., 2003). Thus, butterfly abundance and community composition can provide useful feedback on how grazing affects other grassland‐dependent taxa (Debinski et al., 2011; Moranz et al., 2012; Ravetto Enri et al., 2017). Global pollinator declines threaten ecosystem stability and agricultural production (Grixti et al., 2009; Potts et al., 2010), heightening the importance of understanding how grazing management affect pollinator species and the resources on which they depend.

This study assesses how two different domestic herbivores affect the abundance and diversity of butterflies and floral resources in a landscape managed with patch‐burn grazing. This investigation is especially relevant for the Northern Great Plains, where pollinators of conservation concern such as the regal fritillary (*Speyeria idalia*) and the yellow‐banded bumble bee (*Bombus terricola*) overlap with extensive livestock production. Cattle and sheep production are major economic enterprises in the Northern Great Plains (Montana, Wyoming, Colorado, North Dakota, South Dakota, and Nebraska), resulting in $21.7 billion in cattle sales, $276 million in sales from sheep meat, and an additional $12.7 million from wool production in 2017 (USDA NASS, 2019). Our specific objectives are as follows: (1) evaluate the differences between grazer species (sheep or cattle) on floral resource abundance and richness; (2) quantify butterfly community composition and individual species’ densities in landscapes grazed by sheep or cattle managed within a patch‐burn grazing framework.

## 2 METHODS

### 2.1 Site description

We conducted this research in southwest North Dakota at North Dakota State University's Hettinger Research Extension Center (HREC). During the study (2017–2019), the average temperatures were 12°C in May, 18°C in June, 21.9°C in July, and 19.3°C in August (NDAWN, 2019a). Thirty‐year average precipitation for May–August is 25.1 cm (NDAWN, 2019a). However, over the course of the three seasons of sampling, May–August precipitation ranged from 11.2 cm in 2017, to 22.7 cm in 2018, and 33.3 cm in 2019 (Figure S1, NDAWN, 2019b).

Our study sites are former Conservation Reserve Program fields, planted with Natural Resource Conservation Service Conservation Planting 1 (CP1) “introduced grasses” in the late 1980s (Geaumont et al., 2017). Species established under the CP1 planting included intermediate wheatgrass (*Elymus hispidus* [P. Opiz] Melderis), alfalfa (*Medicago sativa* L.), crested wheatgrass (*Agropyron cristatum* [L.] Gaertn), and yellow sweet clover (*Melilotus officinalis* [L.] Lam.) (Geaumont et al., 2017). While alfalfa and sweet clover are still the most dominant forbs, yarrow (*Achillea millefolium* L.), and common bindweed (*Convolvulus arvensis* L.), field pennycress (*Thlaspi arvense* L.), and herb sophia (*Descurainia sophia* (L.) Webb ex Prantl) were also common.

### 2.2 Experimental design

We conducted research in six, 65‐ha pastures, with three pastures grazed by sheep and three grazed by cattle. Two cattle and two sheep pastures were located 7 km west of Hettinger, ND (site 1, site 2), and the other cattle pasture and sheep pasture were 3 km south of Hettinger (site 3). We weighed and sorted animals to target a moderate stocking rate of 0.5–0.6 ha/AUM (animal unit month) in all pastures (Spiess et al., 2020). We stocked three pastures with cattle (23–30 cow‐calf pairs/pasture) and three with sheep (168–173 ewes/pasture). Previous to the study, land use varied across pastures with areas idle, hayed, or grazed season long (Figure S2). We randomly assigned grazer treatments to each pasture. An analysis of pre‐treatment vegetation community composition shows that forb composition varied between sites and pastures but was not significantly different between pastures assigned to cattle and those assigned to sheep (Figure S3). Animals grazed pastures from May until September. Each pasture was divided into quarters, delineated by a 20’ fire break disked to mineral soil; however, only the exterior of the pasture was fenced allowing for livestock movement across the entire management unit. We burned one quarter of each pasture annually during the dormant season (i.e., four year fire‐return interval), similar to other semi‐arid patch‐burn grazing experiments (Augustine & Derner, 2014; Vermeire et al., 2004).

### 2.3 Data collection

#### 2.3.1 Line transect distance sampling for butterflies

We sampled butterflies from late May to mid‐August of 2017–2019 with three sampling periods per season to quantify butterfly community composition and individual species’ densities. Three sampling periods maximizes detections of species with varying flight periods and voltinism. There were 12, 100‐m butterfly transects per pasture, three in each burn unit, for a total of 72 butterfly transects. To maximize butterfly detections and to minimize variation between surveys, sampling occurred between 08:00 h and 17:30 h as long as temperatures were between 18.3°C and 35.5°C, sustained winds <20 km/h, and cloud cover was <50% (Harmon‐Threatt & Hendrix, 2015; Moranz et al., 2012). We used line transect distance sampling (LTDS) to measure the density of butterflies while accounting for imperfect detection (Brown & Boyce, 1998; Buckland et al., 2001). We conducted LTDS surveys by walking 100‐m transects at an approximate rate of 10 m/min and recorded all butterfly species on either side of the transect, as well as the perpendicular distance from the transect.

#### 2.3.2 Floral and vegetation surveys

After each butterfly survey (i.e., three times per season), we counted all flowering ramets within 1 m of either side of the transect to quantify how different grazers affected floral resources (Shepherd & Debinski, 2005). Flowering ramets were identified to species. Additionally, once per season, we recorded vegetation structural characteristics and vegetation composition along each transect. We collected vegetation data every 10 m on both sides of the 100‐m transect for 20 sampling points per transect. We measured vegetation structure (visual obstruction) with a Robel pole marked in 0.25‐dm increments (Robel et al., 1970) with four visual obstruction readings at each point. The observer also recorded the tallest standing live and standing dead vegetation at each sampling point. Finally, we assessed vegetation composition by species, and percent cover of standing litter, ground litter, and bare ground at each sampling point using a 0.5‐m^2^ frame and the Daubenmire (1959) cover classification (0–5%, 5–25%, 25–50%, 50–75%, 75–95%, 95–100%) and measured litter depth inside each corner of the 0.5‐m^2^ frame.

### 2.4 Data analysis

#### 2.4.1 Effects of grazer species and precipitation on floral characteristics

We performed all statistical analysis in the R statistical environment (R Core Team, 2018). We used general linear mixed‐effect models (GLMMs) using the *glmmTMB* package (Brooks et al., 2017) to assess how grazer treatment and year affected floral attributes. Exploratory analysis revealed strong year effects on both floral and butterfly data. As a result, we summarized data for each transect each year (transect‐year) in order to incorporate year as a variable in our analyses. All subsequent floral variables are based on observations per transect‐year. We summarized the floral data for each transect across the three visits per year using maximum annual counts (McGranahan et al., 2013). We used maximum instead of average to summarize the full flowering potential during the growing season and to avoid any chance of double‐counting flowering stems between sampling periods. Floral abundance was the sum of each species’ maximum flowering stems for each transect‐year. Floral richness was a count of the number of species present for each transect‐year. We used Simpson's Diversity Index to calculate floral diversity for each transect‐year.

We fit GLMMs with grazer plus year as the fixed effects and ran type II ANOVAs to determine whether grazers influenced floral abundance, floral richness, or floral diversity. To account for spatial non‐independence, we nested transect in site as the random effect. A negative binomial distribution best fit our floral abundance data. A Poisson distribution best fit our richness data. Although Simpson's diversity ranges include zero to one, a beta distribution best fit the data; to meet the assumptions of a beta distribution (0 < *y* < 1), we used the transformation suggested by (Smithson & Verkuilen, 2006): *y″* = [*y* (*N* – 1) + 0.5]/*N*, where *y* is the response variable and *N* is the sample size to transform the floral diversity data. We selected distributions for each model by using “descdist” from *fitdistrplus* to assess the skewness and Pearsons's kurtosis values for the response variable (Delignette‐Muller & Dutang, 2015). We used type II ANOVAs to test for significant differences between cattle and sheep. To get pairwise comparisons between grazers for each year, we used package *emmeans* (Lenth, 2019) to compute estimated marginal means for these models. We obtained pairwise comparisons for the estimated marginal means using the “lsmeans” functions from *emmeans* (Lenth, 2019), which use a significance level of *α* = 0.05 and the Tukey method for comparing a family of six estimates.

We calculated the effect sizes of grazer (sheep vs cattle), our dry year versus our near‐average year (2017 vs. 2018), and our wet year versus our near‐average year (2019 vs. 2018) to compare the relative effects of precipitation versus grazer species. We obtained confidence intervals for the pairwise comparisons from the previous GLMMs using the “confint” function (R Core Team, 2018).

#### 2.4.2 Effect of time since fire on floral characteristics

Given our uneven sample size in time since fire intervals, we limited our analysis to recently burned‐unburned comparisons. Each year, we burned half of each pasture. This study covered the first three years of the treatment; hence, only three of the four burn units in each pasture received a fire treatment and one unit in each pasture remained unburned. Our hesitation in comparing all four time since fire intervals (unburned, recently burned, one year since fire, two years since fire) is that 2019 was the only year that contained two year since fire observations. Since there was strong yearly variation in floral characteristics, it seemed preferable to use data from the patch that remained unburned throughout the study and the most recently burned unit in each pasture to assess whether there was an effect of fire on floral variables. We created a model for each floral characteristic (floral abundance, floral richness, floral diversity) in “glmmTMB” using burned/unburned as the main effect. Again, we used “descdist” from *fitdistrplus* to assess the skewness and Pearson's kurtosis values for the response variable and to select a distribution (Delignette‐Muller & Dutang, 2015). For floral abundance, we used a Poisson distribution; for floral richness, we used a negative binomial; and for floral diversity, we used a beta distribution and Smithson and Verkuilen's weighted average to transform diversity values (Smithson & Verkuilen, 2006). To account for repeated measures, site differences, and grazer effects, we included pasture and year as crossed random effects. We used type II ANOVAs to test for significant differences between burned and unburned. Although comparing unburned to recently burned precluded us from assessing if the intermediate time‐since‐fire intervals were the most ecologically beneficial, we wanted to have some indication of the effects of fire treatments on floral variables.

#### 2.4.3 Effect of grazer species on butterflies

Similar to our floral characteristic analyses, we created GLMMs with grazer plus year as the fixed effects and ran type II ANOVAs to determine whether grazers influenced butterfly abundance, butterfly richness, or butterfly diversity. We did not expect grazers to have a direct effect on butterflies, however, looking at grazer effects may reveal patterns not captured by our floral analyses. Again, we selected distributions for each model by using “descdist” from *fitdistrplus* (Delignette‐Muller & Dutang, 2015). A lognormal distribution best fit our butterfly abundance, butterfly richness, and butterfly diversity data. We used *emmeans* to calculate pairwise comparison using a significance level of *α* = 0.05 and the Tukey method for comparing a family of 6 estimates.

#### 2.4.4 Butterfly community analysis

We used the *vegan* package (Oksanen et al., 2019) to explore relationships between grazer effects, floral resources, time since fire (TSF), and site characteristics on butterfly community composition using non‐metric multidimensional scaling (NMDS) ordinations. To assess butterfly community composition using ordination, we summarized butterfly data as the maximum number of observations for each species per transect‐year. Our final community dataset contained 11 species that ranged from 21 to 5463 detections. We created our ordinations using the “metaMDS” function in *vegan*. We used the Canberra metric for the butterfly community ordination because it more accurately represents the dissimilarity space and resulted in lower stress scores (Kindt & Coe, 2005). Our ordination had four axes (k=4) and a stress value of 0.124.

We assessed the effects of grazer, year, time since fire, and floral characteristics on the butterfly community via the “envfit” function in *vegan* (Oksanen et al., 2019). To account for effects of inherent spatial heterogeneity, pasture (*n* = 6) was incorporated as a random effect (strata) within “envfit”. We grouped plant species into the following functional groups: native forbs, introduced forbs, native grass, introduced grass, and native shrub. Origin of plants (non/native) was verified using the NRCS PLANTS database (USDA, 2020). We incorporated site characteristics by taking the average values for plant functional groups, ground cover, bare ground cover, litter cover, litter depth, visual obstruction, tallest live and tallest dead plants for each transect‐year. Using transect‐year averages for site/vegetation metrics standardized these observations to the same scale as our butterfly data. Additionally, ordinations struggle to cluster sites if there are many zeros in the dataset; averaging to the transect‐year and using functional groups for plants minimized the number of zeros in dataset allowing for ordination convergence. We then assessed how much variance in the ordination was explained by grazer, year, time since fire, or vegetation characteristics with a type II PERMANOVA.

#### 2.4.5 Butterfly density estimates

We assessed the influence of grazing treatments on individual species’ densities by calculating corrected butterfly densities for the five most abundant species (92.3% of observations) using package *unmarked* (Fiske & Chandler, 2011). This method enables us to incorporate detection probability into our analysis to get a corrected density estimate (Buckland et al., 2001). Of the 27 species we observed during surveys, five species (*Colias philodice*, *Plebejus melissa*, *Vanessa cardui*, *Pontia protodice*, *Colias eurytheme*) had sufficient observations each year (50+) to get robust predicted densities (Buckland et al., 2001). We determined which detection function best described the data for each of the five species using the half‐normal, hazard rate, exponential, and uniform key functions, and ranked candidate models using Akaike information criterion (AIC). Models with ΔAIC ≤2 were considered to have the same explanatory value (Burnham & Anderson, 2003). After selecting the best key function, we then created univariate models to test effects of year, floral abundance, floral richness, and floral diversity on densities for each butterfly species. We standardized floral abundance and richness by subtracting the mean from individual values and dividing by standard deviation. Unsurprisingly, Pearson's correlation coefficient showed that floral richness and diversity were correlated. We kept both variables, but assessed them in separate models to examine whether butterflies were influenced by richness or evenness in addition to diversity. We developed model sets, which included a null model, year only, transect‐level floral characteristics, and transect‐level floral characteristics with year as additive and as an interaction. We ranked models using AIC and we considered models with ΔAICc ≤2 to have the same explanatory power about species density (Burnham & Anderson, 2003). We computed estimated densities and 95% confidence intervals based on the most competitive model for each species and graphed the resulting relationship.

## 3 RESULTS

We counted 196,806 flowering ramets of 95 different plant species from 2017 to 2019. Alfalfa accounted for 70.4% of floral counts and native forbs were 8%. Floral abundance and richness increased each year; we counted 35,966 flowering stems of 37 species in 2017, 57,326 ramets of 54 species in 2018, and 103,514 ramets of 66 species in 2019. We observed 13,783 butterflies across the three seasons. Butterfly abundance increased each year with 2646 observations in 2017, 4722 in 2018, and 6415 in 2019. Species richness also increased as the study progressed with 17 species observed in 2017, 20 in 2018, and 26 in 2019. *Colias philodice*, a disturbance‐ and agricultural‐tolerant species was the most abundant, accounting for 61.3% of observations (8449 detected). The top five most abundant species (including *C*. *philodice*) were all agricultural‐tolerant, diet generalist species whose caterpillars can subsist on non‐native/weedy mustards or alfalfa: *Plebejus melissa* (21.3% of observations), *Vanessa cardui* (4.3% of observations), *Pontia protodice* (3.7% of observations), *Colias eurytheme* (1.7% of observations). Species of conservation concern such as *Speyeria idalia* (regal fritillary) and *Danaus plexippus* (monarch) represented <0.5% of observations (Table S1). Fifty‐nine percent of butterfly observations occurred in cattle pastures, which encompassed 27 out of 28 observed species. Sheep pastures had lower butterfly richness with 23 species (Table S1).

### 3.1 Effect of grazer species and precipitation on floral resources

**FIGURE 1:**
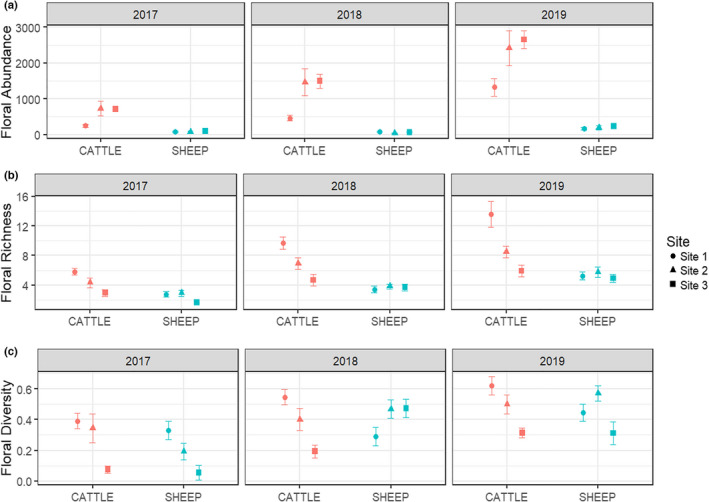
Mean floral abundance (a), floral richness (b), and floral diversity (c) by year and grazer in each pasture for 2017–2019 in Post‐Conservation Reserve Program grasslands in southwest North Dakota, USA. Cattle pasture values are displayed in red and sheep in blue, error bars represent standard error. Sites represent our pasture replicates

Pastures grazed by cattle had significantly more flowers than those grazed by sheep (floral abundance: *χ* ^2^ = 184.08, *df* = 1, *p* < .001, Figure 1). There was no significant difference between floral abundance in sheep pastures in 2017 and 2018 (sheep 2017 vs. sheep 2018: *df* = 207, *p* = .115), but all other pairwise comparisons between grazers‐years were significant at *α* = 0.05 level (Figure S4). Floral richness was significantly higher each year within grazer treatments for both cattle and sheep (Figure S5) and cattle pastures always had more floral richness than sheep pastures (floral richness: *χ* ^2^ = 44.6, *df* = 1, *p* < .001, Figure 1). Floral diversity was not significantly different between grazers (floral diversity: *χ* ^2^ = 1.92, *df* = 1, *p* = .166, Figure 1).

**FIGURE 2:**
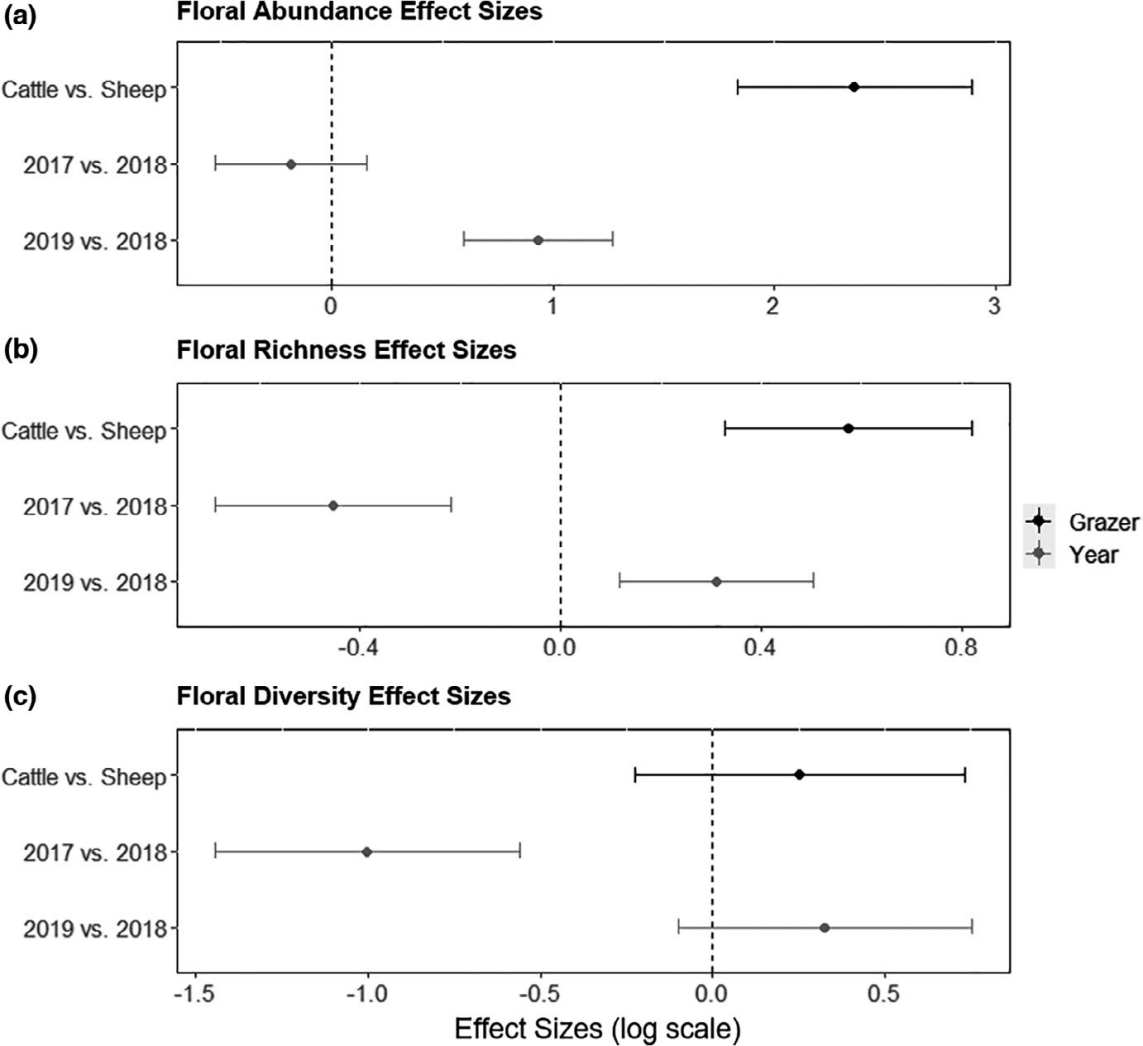
Effects of grazer species (Cattle vs. Sheep), dry year compared to near‐average year (2017 vs. 2018), wet year compared to near‐average year (2019 vs. 2018) on floral abundance (a), floral richness (b), and floral diversity (c) in Post‐Conservation Reserve Program grasslands in southwest North Dakota, USA. Points are standardized effect sizes ±95% CI. An effect size is considered significant when its CI does not include zero

There was a strong year effect on floral abundance in cattle pastures, but floral availability in sheep pastures remained low regardless of year (Figure 1). Both cattle grazing and a wet year (2019 vs. 2018) had a positive effect on floral abundance, but effect sizes showed cattle grazing compared to sheep grazing had a much larger effect on floral abundance than precipitation (Figure 2a, Cattle vs. Sheep, *d’* estimate = 2.76, CI.95 = 2.29–3.23; 2019 vs. 2018, *d’* estimate = 0.94, CI.95 = 0.59–1.28). The effects of grazer species and precipitation were smaller for floral richness; grazer and a wet year had a positive influence and a drought year had a negative effect (Figure 2b, Cattle vs. Sheep, *d’* estimate = 0.72, CI.95 = 0.52–0.92; 2017 vs. 2018, *d’* estimate = −0.25, CI.95 = −0.47 to −0.03; 2019 vs. 2018, *d’* estimate = 0.41, CI.95 = 0.22–0.60). Unlike floral abundance and richness, grazer species and a wet year did not have a significant effect on floral diversity. Instead, lower precipitation had a medium to strong negative effect on floral diversity (Figure 2c, Cattle vs. Sheep, *d’* estimate = 0.85, CI.95 = 0.47–1.23; 2017 vs. 2018, *d’* estimate = −0.10, CI.95 = −0.45 to 0.25; 2019 vs. 2018, *d’* estimate = 0.81, CI.95 = 0.45–1.17).

### 3.2 Effect of time since fire on floral characteristics

Floral abundance was significantly different between recently burned and unburned units (Floral abundance: *χ* ^2^ = 6068.9, *df* = 1, *p* < .001). There were more flowers in unburned units than recently burned units (*β*_unburned_ = 6.05, LCI = −5.13, UCI = 6.98; *β*_burned_ = 6.59, LCI = −5.62, UCI = 7.53). Floral richness was not significantly different between unburned and recently burned units (floral richness: *χ* ^2^ = 1.188, *df* = 1, *p* = 0.276), nor was floral diversity (floral diversity: *χ* ^2^ = 6.1.722, *df* = 1, *p* = .6319).

### 3.3 Effect of grazer species on butterflies

**FIGURE 3:**
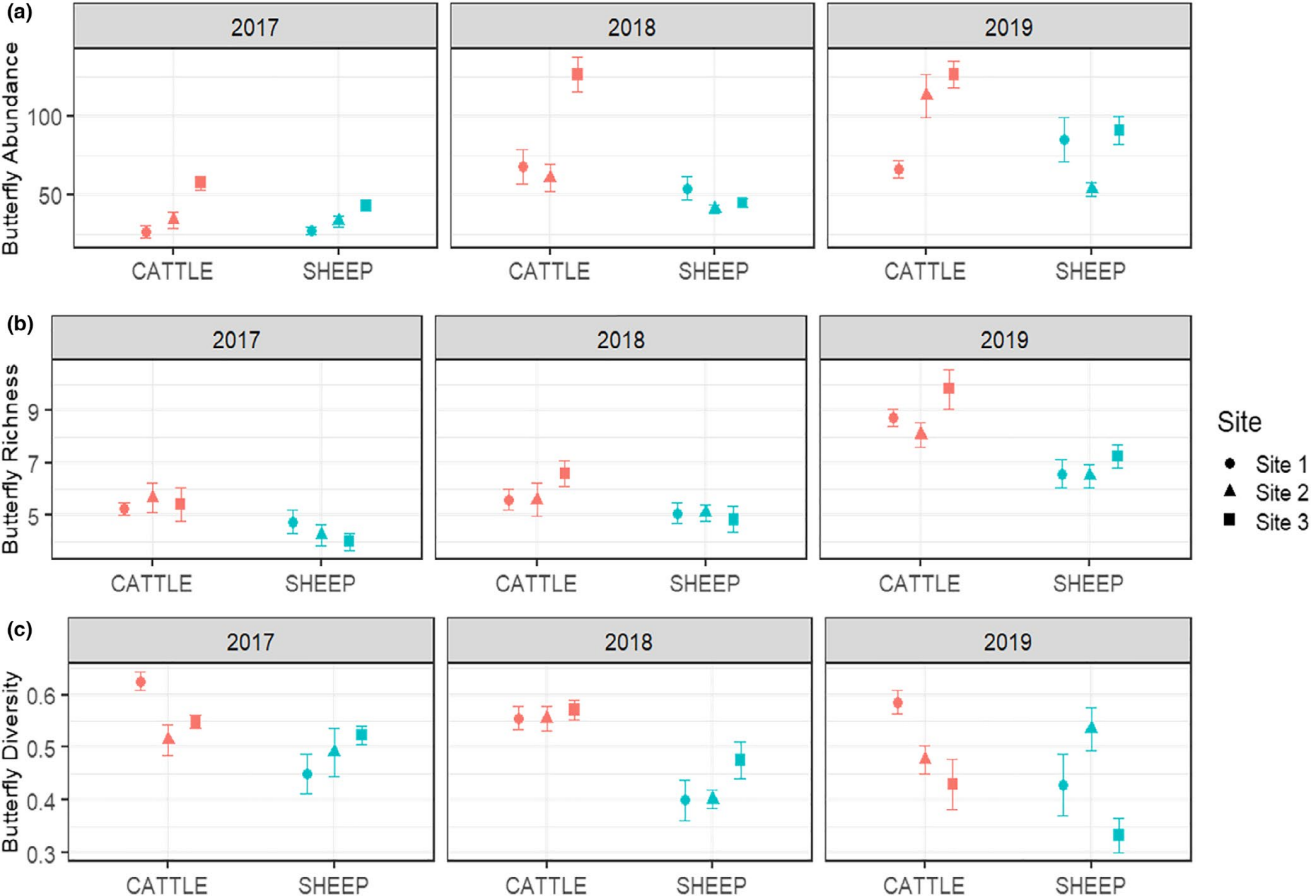
Mean butterfly abundance (a), butterfly richness (b), and butterfly diversity (c) per transect by year and grazer in each pasture for 2017–2019 in Post‐Conservation Reserve Program grasslands in southwest North Dakota, USA. Cattle pasture values are displayed in red and sheep in blue, error bars represent standard error. Sites represent our pasture replicates

Overall, pastures grazed by cattle had significantly more butterflies, butterfly species, and higher butterfly diversity than those grazed by sheep (butterfly abundance: χ^2^ (1) = 15.054, *p* < .001; butterfly richness: χ^2^ (1) = 25.94, *p* < .001, butterfly diversity: χ^2^ (1) = 25.315, *p* < .001, Figure 3). In 2017, there was not a significant difference in butterfly abundance between grazers (cattle 2017 vs. sheep 2017: *df* = 207, *p* = .997, Figure S7). In 2018 and 2019, there were significantly more butterflies in cattle pastures (*p* < .0.001, Figure S7). All cattle sites in 2018 had more butterflies than the sheep sites; in 2019, two of the three cattle sites had more butterflies (Figure 3). There was evidence for a significant grazer‐year interaction affecting butterfly abundance (Grazer: year: χ^2^ (2) = 12.840, *p* = .0016). For butterfly richness per transect, there was no significant difference between cattle and sheep pastures in 2018 (cattle 2018 vs. sheep 2018: *df* = 207, *p* = .250, Figure S8). There was not a significant grazer‐year interaction (Grazer: year: χ^2^ (2) = 1.453, *p* = .4836). Across all years, butterfly diversity was significantly higher in cattle pastures and there was not a significant grazer‐year interaction (Grazer: year: χ^2^ (2) = 2.470, *p* = .2908, Figure S9).

### 3.4 Butterfly community analysis

**FIGURE 4:**
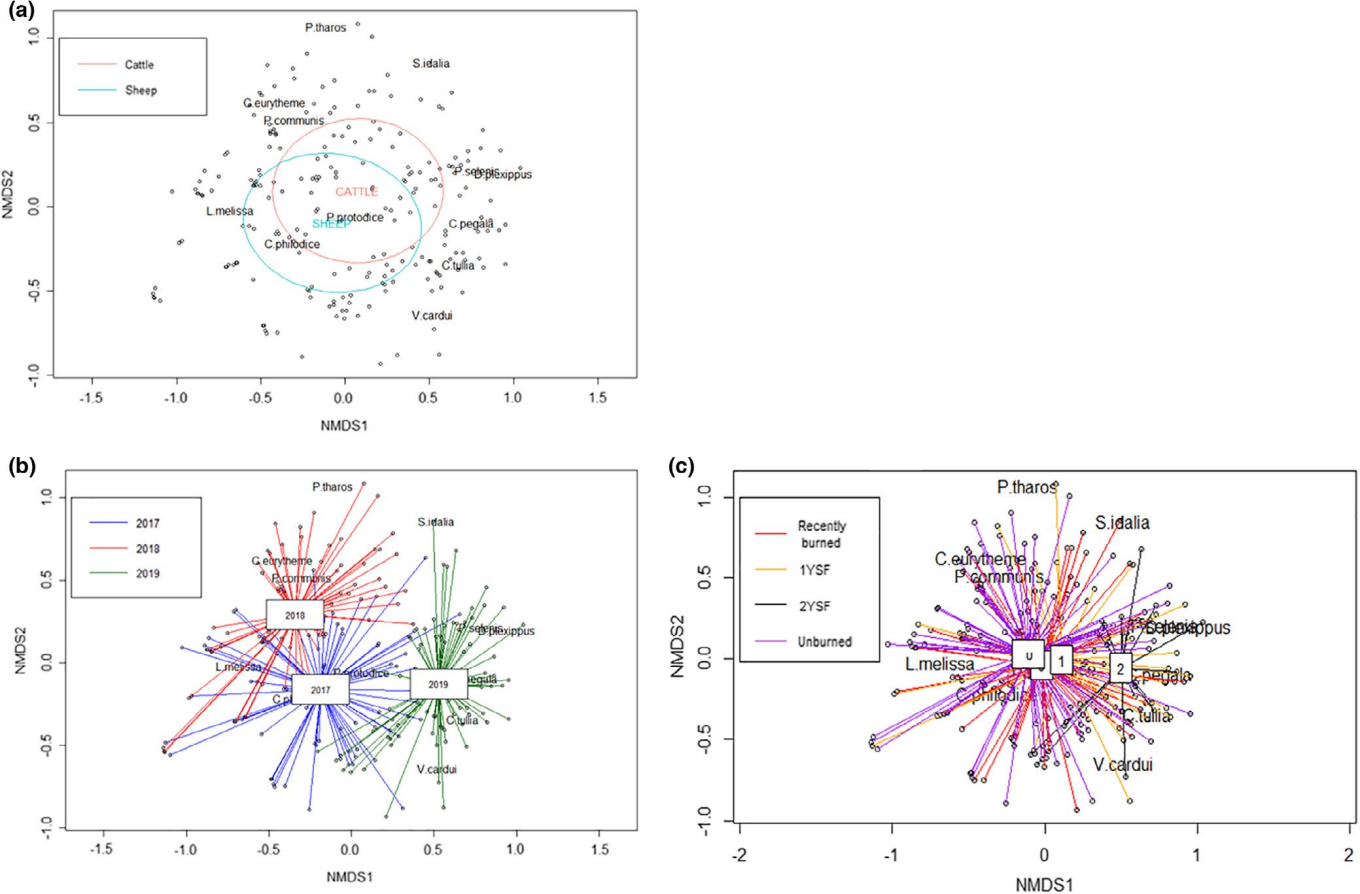
Butterfly community NMDS ordination showing grazers (a), species and year (b), and time since fire (c) groupings for 2017 – 2019 at Hettinger Research Extension Center (k = 4, stress = 0.124). Panel a: Grazer had minimal association with variation in the butterfly community (pseudo‐*F* ratios, *p* < .01, *R* ^2^ = 0.02). Panel b: Year was associated with distinct patterns of variation in butterfly communities (pseudo‐*F* ratios, *p* < .01, *R* ^2^ = 0.19). Panel c: Overlap in butterfly communities based on time since fire intervals: never burned (U), recently burned (0), and one year since fire (1). Two years since fire (2) is separate, but likely an artifact of limited sample size and data only from 2019

Grazer species, TSF, floral attributes, and site characteristics showed minimal association with patterns in the butterfly community, with only year associated with distinct dissimilarities in butterfly community. We found considerable overlap in butterfly communities regardless of grazer treatment (Figure 4a, stress = 0.124, *k* = 4). Grazer species was associated with less than 2% of the variance in the butterfly community (pseudo‐*F* ratios, *p* < .01, *R* ^2^ = 0.02). Year was most strongly associated with distinct patterns in the butterfly community (Figure 4b; pseudo‐*F* ratios, *p* < .01, *R* ^2^ = 0.19). Time since fire intervals for unburned, recently burned, and one‐year post‐fire were clustered around the origin, meaning there was minimal variation between sites based on those time since fire (Figure 4c; pseudo‐*F* ratios, *p* < .01, *R* ^2^ = .04). Two years since fire was further from origin, however, 2019 was the only season with 2 years since fire data, so that result is likely an artifact of limited sample size rather than a biological response to two years since fire. To see whether year was overshadowing effects of time since fire, we created separate NMDS ordinations for each year, but within each year, time since fire intervals overlapped and were not associated with a distinct pattern in the butterfly community (Figure S10). All floral attributes and site characteristics were significant (pseudo‐*F* ratios, *p* < .001), but were associated with minimal variation in butterfly communities (*R* ^2^ = 0.02–0.07).

### 3.5 Individual species density estimates

**FIGURE 5:**
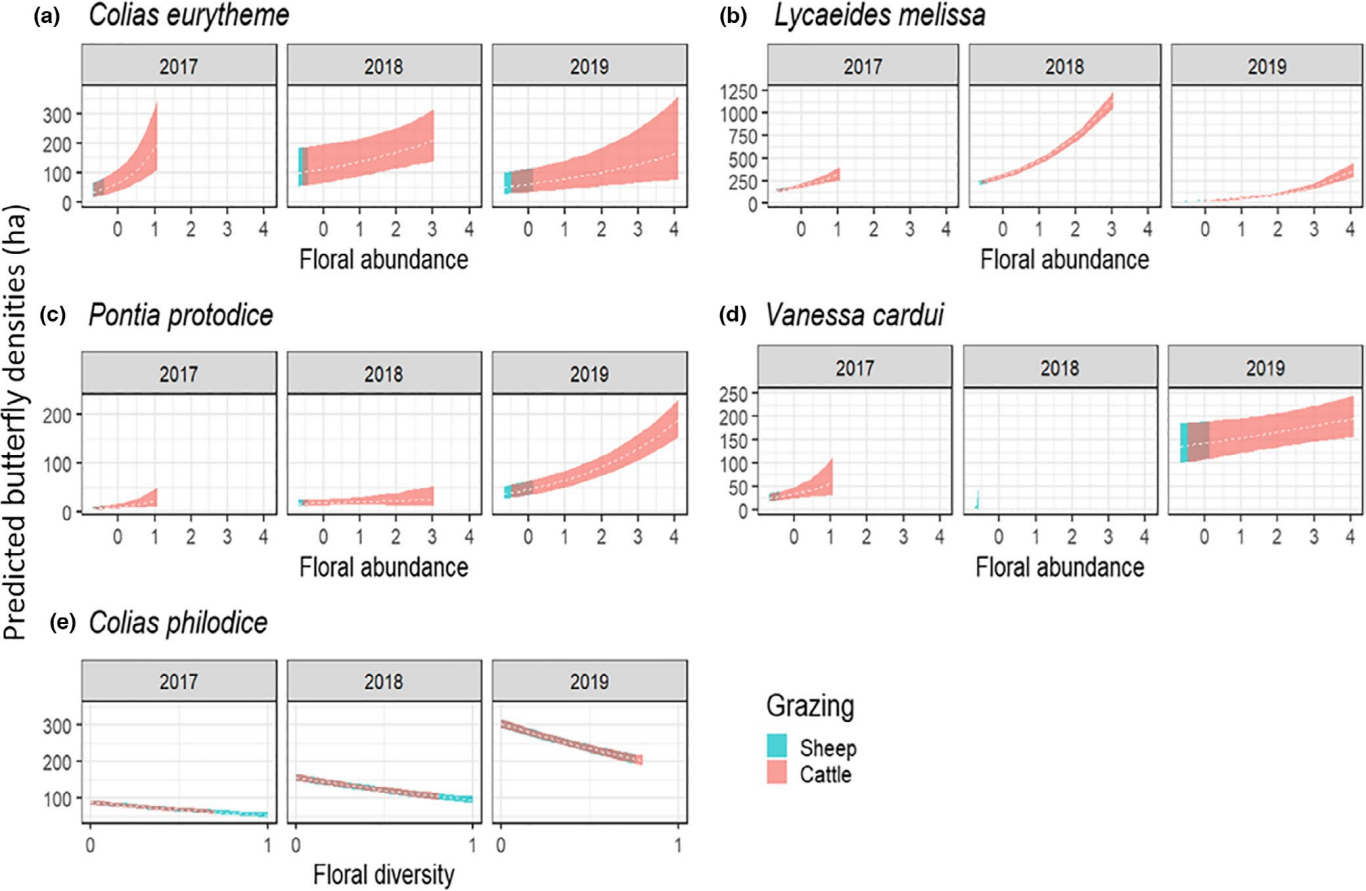
Predicted density estimates for butterfly species based on average floral attributes for 2017–2019 at Hettinger Research Extension Center. Standardized floral abundance is shown in panels a, b, c, d, floral diversity in shown in panel e. The dotted line shows the estimated butterfly density, the width of the curve represents the upper and lower bounds of the estimate. We shaded predicted density curves to show the relationship between livestock species and depicted floral abundances or diversities. Blue depicts floral attribute ranges that occurred in cattle pastures and pink shows the same for sheep pastures. Panels a, b, c, d show densities for these butterflies increased with increasing floral abundance. Panel e shows c. philodice is more abundant in pastures with low floral diversity

A model that incorporated floral abundance per transect and an interaction with year was the most competitive model for *V*. *cardui*, *P*. *protodice*, *C*. *eurytheme*, and *L*. *melissa* (Table 1). For all four species, higher floral abundance was associated with higher predicted density (Figure 5). *V*. *cardui* is an irruptive species and while abundant in 2017 and 2019, it was nearly absent in 2018, making it difficult for the model to predict *V*. *cardui* for that year. For *C*. *philodice*, the most competitive model comprised floral diversity per transect with year as an additive interaction (Table 1). Density of *C*. *philodice* was negatively correlated with flowering plant diversity (Figure 5e). For all five butterfly species, their highest predicted densities were associated with the floral abundances or floral diversities observed in cattle pastures.

**TABLE 1:** Top butterfly density model outputs across 2017–2019 at Hettinger Research Extension Center. Most competitive model outputs for the five grassland butterfly species meeting the minimum detection threshold ford density estimation. A model incorporating floral abundance and year interaction was the most competitive for four of the species. Floral diversity with year as an additive factor best described *C*. *philodice* abundance

Model	nPars	AIC	delta AIC	AIC weight	Cumulative weight
*Colias eurytheme*					
Floral abundance * year	8	1395.59	0	9.20E−01	0.92
Floral abundance + year	6	1401.1	5.51	5.80E−02	0.98
*Plebejus melissa*					
Floral abundance * year	8	3245.74	0	1.00E+00	1
Floral abundance + year	6	3257.96	12.22	2.20E−03	1
*Pontia protodice*					
Floral abundance * year	9	2610.56	0	7.60E−01	0.76
Floral abundance + year	7	2612.85	2.3	2.40E−01	1
*Vanessa cardui*					
Floral abundance * year	9	2641.56	0	6.40E−01	0.64
Floral abundance + year	7	2642.93	1.37	3.20E−01	0.97
Floral diversity + year	7	2649.68	8.12	1.10E−02	0.98
*Colias philodice*					
Floral diversity + year	6	−9677.01	0	7.60E−01	0.76
Floral diversity * year	8	−9674.61	2.4	2.30E−01	0.99

## 4 DISCUSSION

Agricultural intensification, specifically the conversion of grasslands to row crops, threatens the ability of native species to persist on the landscape (Deguines et al., 2014; Sala et al., 2000; Tscharntke et al., 2005). By utilizing semi‐natural areas such as former CRP lands for grazing, managers and conservations can maintain perennial cover that grasslands organisms need to persist. Understanding how management choices and precipitation affect floral resources and native pollinators on these areas enables managers to develop conservation strategies to promote biodiversity. Our results indicate that compared to cattle grazing, sheep grazing resulted in lower floral richness and much lower floral abundance. This trend aligns with studies that also found sheep grazing resulted in lower floral richness and abundance as compared to pastures grazed by cattle (Carvell, 2002; Dumont et al., 2011; Jerrentrup et al., 2015; Ravetto Enri et al., 2017). There was also lower butterfly richness and slightly lower abundance in sheep pastures. However, our community analysis did not show a significant effect of grazer or time since fire on butterfly composition, likely due to the homogeneity of the butterfly and plant communities (Bendel et al., 2018). Additionally, species of conservation concern such as *Danaus plexippus* and *Speyeria idalia* comprised <0.5% of butterfly observations. High variability in annual rainfall may have overshadowed some of our expected effects of fire and grazing (Lanta et al., 2014). Our results indicate that higher precipitation and cattle grazing can improve floral and butterfly species richness and abundance in low‐diversity grasslands, but in general, these grasslands would benefit from proactive measures to restore native forb diversity and abundance to support native pollinator communities.

Sheep grazing resulted in lower floral abundance and richness compared to cattle in our experimental pastures, suggesting that moderately stocked, season‐long sheep grazing (even in a patch‐burning system) is less beneficial to pollinator conservation than cattle grazing. Similar to other studies, we found lower floral and butterfly abundance and richness in sheep pastures compared to pastures with cattle (Carvell, 2002; Dumont et al., 2011; Jerrentrup et al., 2015; Ockinger & Smith, 2007; Ravetto Enri et al., 2017). It is unclear whether changing the stocking rate would improve floral conditions. A high stocking rate can reduce sheep selectiveness, but can be more deleterious to flower frequency than a low stocking rate or no sheep (Lanta et al., 2014). Lower stocking rates may reduce overall pressure, but sheep selectivity can still shape grassland community composition (Pittarello et al., 2017).

Floral abundance and richness responded positively to a wet year and floral richness and diversity responded negatively to a drought. High annual variability in precipitation is common in grasslands (Lauenroth & Sala, 1992) and affects above‐ground primary productivity and plant species composition (Heisler‐White et al., 2009; Knapp & Smith, 2001; Lauenroth & Sala, 1992; Silvertown et al., 1994). Precipitation variability can enhance plant community diversity (Knapp et al., 2002; Silvertown et al., 1994). We saw similar trends in our study with increased floral richness with increased precipitation, however, the majority of the increase in forb expression occurred in plants that were already dominant or common at our sites (alfalfa, sweet clover). Our results highlight the important role of precipitation in regulating forb expression and community composition in grassland systems.

We expected that patch‐burn grazing would reduce forb selection in sheep pastures, possibly to the extent that floral metrics in cattle and sheep pastures would be similar (Allred et al., 2011; Archibald et al., 2005). However, we found that floral availability in sheep pastures was orders of magnitude lower than that of cattle pastures, suggesting that patch‐burn grazing in low‐diversity grasslands did not ameliorate the effects of sheep grazing. We did find that unburned units had greater floral abundance than recently burned units, which may suggest that grazers were spending less time grazing in unburned units compared to recently burned ones. Several studies have documented a post‐fire flush of forbs due to increased blooming duration or number of ramets (Mola & Williams, 2018; Wrobleski & Kauffman, 2003). If we saw greater forb abundance in unburned pastures, perhaps that is an indication that grazers were focusing their attention on the recently burn patches, but not enough to close the gap between grazers.

Although there was minimal variation in ordination space between time since fire intervals and the butterfly community, this lack of a trend suggests that fire was not detrimental to the butterfly community. We did not observe a strong association between species of concern and unburned units, which would have suggested that those species preferred the habitat characteristics associated with no fire or longer fire intervals. We burned three‐fourth of each pasture by the end of this study, but a full application of patch‐burn grazing would mean that each unit in the pastures have received fire at least once (Allred, Fuhlendorf, Engle, et al., 2011; Archibald et al., 2005; Fuhlendorf & Engle, 2004). More years of fire application would also result in greater sample size for each time since fire benchmark (recently burned, 1, 2, 3 years since fire) and more statistical power to determine how time since fire affects specific species and the pollinator community (McCullough et al., 2019; Moranz et al., 2012, 2014; Potts et al., 2003). Overall butterfly abundance and richness were higher in cattle pastures, but our community analysis did not find a strong association between grazer treatment or site characteristics and patterns of variation in the butterfly community. This contrasts previous studies that found vegetation characteristics strongly affect butterfly communities (Davis et al., 2007; Debinski et al., 2011; Pöyry et al., 2006; Sjödin et al., 2008; Vogel et al., 2007). Our results may differ because several of these studies occurred on remnant prairie sites with higher native flower presence where habitat specialists comprised a higher proportion of the butterfly community (Davis et al., 2007; Debinski et al., 2011; Vogel et al., 2007). Additionally, the high abundance of *C*. *philodice* and *P*. *melissa* (82.6% of all observations) at all sites may have limited the ability of the ordination to discern differences between pastures. Additionally, the majority of these studies occurred in wetter climates (Iowa, Sweden, Finland), which may influence relationships between site characteristics and butterfly community variation. Our study experienced high precipitation variation and showed low diversity in plant and butterfly communities.

The lack of grassland‐obligate and specialist butterflies at our sites suggests that older, low‐diversity CRP plantings may not be providing species of concern with the resources that they need. During our study, less than 3% of observations were butterfly species with host–plant specialists (diet breadth limited to only one genus) and less than 0.5% of observations were *Speyeria idalia* and *Danaus plexippus*, two host–plant specialists of conservation concern. A study comparing butterfly communities between marginal and intact habitat have also noted much lower densities of *S*. *idalia* on marginal grasslands (Farhat et al., 2014). They found over five times as many *S*. *idaliai* per kilometer on intact tallgrass prairie compared to field edges, roadside, or ditches. Studies comparing native bee communities in marginal versus intact grasslands reveal similar trends, with marginal areas supporting fewer bees and bee species and fewer flowers than intact grasslands (Hopwood, 2008; Kwaiser & Hendrix, 2008; Wood et al., 2017).

Agri‐environmental schemes such as the CRP are generally thought to be important refuges for wildlife within agricultural landscapes (Jones‐Farrand et al., 2007). Our results suggest that older CRP plantings, especially those planted with CP1 “introduced grasses”, may only be supporting a subset of the butterfly community. The dominance of generalist species at our sites suggests either they are already showing the after‐effects of biotic homogenization due to agricultural intensification, or butterfly diversity is still present on the landscape, but older CRP sites may not be providing the resources needed for a diverse butterfly community (Börschig et al., 2013; Ekroos et al., 2010; Farhat et al., 2014). These results are salient for landscape‐level modeling, which often assumes that perennial cover such as CRP is providing the resources that native pollinators need (Otto et al., 2016). Modeling inherently involves making assumptions and aggregating land‐cover classes; however, the results of our study show that models that do not differentiate between newer pollinator‐friendly plantings and older non‐native grass plantings may overestimate the amount of pollinator resources available on the landscape. Newer CRP practices such as CP42 “Pollinator Habitat Initiative” incentivize the use of wildflower and forb seed mixes instead of just grasses. However, the benefits of this improved seeding mix may be limited by low and/or patchy enrollment, which may not provide sufficient area or connectedness for resilient pollinator populations (Ritten et al., 2017; Scheper et al., 2013; Tscharntke et al., 2005). Improving pollinator resources in agricultural landscapes requires incentivizing not only pollinator‐friendly plantings or pasture improvements but also rewarding landowners who employ these practices on sizeable and/or connected plots (Ritten et al., 2017).

Due to logistical constraints, our study did not have a paired control, which would have allowed us to parse the extent to which precipitation seems to be the most salient factor influencing floral and butterfly richness and abundance in ungrazed and/or unburned pastures. However, the main focus of this study was to compare effect of cattle grazing versus sheep grazing in a patch‐burn management system. In keeping with the applied nature of our research, landowners rarely leave pastures idle. The mixed land‐use history of the pastures could potentially be driving trends in floral metrics. However, grazers were assigned randomly to pastures, site was incorporated into analyses as a random effect, and our figures include how floral and butterfly metrics varied by site and grazer. The trends in floral abundance and floral richness between grazers are stark enough to suggest they are a treatment effect, not a legacy effect. Therefore, we accurately documented changes that may occur as grassland previously enrolled in the CRP are transitioned to grazing lands (Claassen, 2011). We recognize that six, 65‐ha pastures, while “ranch scale” still represent a limited subset of former CRP fields. Our results suggest that more research is needed to assess the extent to which CRP plantings—both older, grass‐heavy seedings, and newer, more forb‐rich mixes—are supporting a diverse community of pollinators.

## 5 CONCLUSIONS

Low‐diversity grasslands need proactive conservation approaches to enhance floral resources that can sustain and improve pollinator populations. As available grassland habitat in the northern Great Plains decreases due to agricultural intensification (Wright & Wimberly, 2013), CRP conversion to grazing lands represents possible refuge for pollinators and other species that cannot survive in row crop monocultures. The integrity of grassland resources and grassland‐dependent wildlife populations in the region depends on recognizing that low‐diversity grasslands may need active intervention and restoration in order to provide sufficient native forb diversity and abundance for native pollinators. Without active interventions like seeding native forbs or reconstructing grasslands, low‐diversity grasslands show minimal trajectory toward the ecological function and plant–insect interactions present in remnant and/or high diversity grasslands (Orford et al., 2016; Woodcock et al., 2012). Additionally, supporting pollinators may require adjusting livestock management practices, such as excluding sheep from areas of the pasture, especially during peak bloom, to enhance floral availability and pollinator abundance and richness.

## CONFLICT OF INTEREST

None declared.

## AUTHOR CONTRIBUTIONS

**Jasmine Cutter:** Data curation (lead); Formal analysis (equal); Investigation (lead); Writing‐original draft (lead); Writing‐review & editing (lead). **Torre Hovick:** Conceptualization (lead); Funding acquisition (lead); Methodology (equal); Project administration (equal); Resources (lead); Supervision (lead); Writing‐review & editing (supporting). **Devan McGranahan:** Conceptualization (equal); Formal analysis (equal); Funding acquisition (supporting); Methodology (supporting); Visualization (equal). **Jason Harmon:** Formal analysis (supporting); Writing‐review & editing (supporting). **Ryan Limb:** Conceptualization (supporting); Funding acquisition (supporting). **Jonathan Spiess:** Formal analysis (supporting); Writing‐review & editing (supporting). **Benjamin Geaumont:** Conceptualization (lead); Funding acquisition (lead); Investigation (supporting); Methodology (lead); Project administration (lead); Supervision (lead); Writing‐review & editing (supporting).

## Supporting information

Appendix S1

## Data Availability

Data used for this analysis are available from figshare Digital Repository: https://doi.org/10.6084/m9.figshare.16875961
