# Supplementary material for: Cattle grazing results in greater floral resources and pollinators than sheep grazing in low‐diversity grasslands
Source: Ecol Evol. 2022 Jan 13;12(1):e8396. doi: 10.1002/ece3.8396 (PMC8809440; doi:10.1002/ece3.8396)
Supplement: Supplementary file 1 — Appendix S1 [file ECE3-12-e8396-s001.docx]

APPENDIX S1. SUPPLEMENTARY MATERIALS FOR CHAPTER ONE

**Table** **S1. Butterfly abundances by year.**

List of butterfly abundances by year and percent of observations in cattle pastures during line-transect distance sampling conducted from 2017-2019 in southwest North Dakota.

| **Butterfly Species** | **2017** | **2018** | **2019** | **Total** | **% in Cattle Pastures** |  |  |
| --- | --- | --- | --- | --- | --- | --- | --- |
|  |  |  |  |  |  |  |  |
| *Colias philodice* | 1406 | 2429 | 4614 | 8449 | 52.7 |  |  |
| *Plebejus melissa* | 894 | 1759 | 286 | 2939 | 74.7 |  |  |
| *Vanessa cardui* | 104 | 1 | 491 | 596 | 51.0 |  |  |
| *Pontia protodice* | 51 | 108 | 349 | 508 | 65.4 |  |  |
| *Colias eurytheme* | 53 | 114 | 71 | 238 | 62.2 |  |  |
| *Cercyonis pegala* | 21 | 22 | 151 | 194 | 51.0 |  |  |
| *Phyciodes selenis* | 2 | 44 | 129 | 175 | 64.0 |  |  |
| *Phyciodes tharos* | 0 | 99 | 44 | 143 | 88.1 |  |  |
| *Coenonympha tullia* | 37 | 14 | 66 | 117 | 73.5 |  |  |
| *Pyrgus communis* | 24 | 49 | 35 | 108 | 71.3 |  |  |
| *Glaucopsyche lygdamus* | 13 | 21 | 38 | 72 | 77.8 |  |  |
| *Pieris rapae* | 15 | 25 | 7 | 47 | 57.4 |  |  |
| *Vanessa atalanta* | 4 | 0 | 34 | 38 | 36.8 |  |  |
| *Danaus plexippus* | 0 | 9 | 20 | 29 | 48.3 |  |  |
| *Speyeria aphrodite* or *Speyeria cybele* | 4 | 2 | 22 | 28 | 55.6 |  |  |
| *Phyciodes batesii* | 4 | 1 | 18 | 23 | 87.0 |  |  |
| *Euptoieta claudia* | 0 | 15 | 6 | 21 | 47.6 |  |  |
| *Speyeria idalia* | 7 | 5 | 9 | 21 | 47.6 |  |  |
| *Polites themistocles* | 5 | 0 | 5 | 10 | 100 |  |  |
| *Vanessa virginiensis* | 2 | 2 | 6 | 10 | 80.0 |  |  |
| *Pholisora catullus* | 0 | 0 | 6 | 6 | 50.0 |  |  |
| *Polites mystic* | 0 | 0 | 5 | 5 | 100 |  |  |
| *Phyciodes cocyta* | 0 | 0 | 4 | 4 | 100 |  |  |
| *Lycaena helloides* | 0 | 3 | 0 | 3 | 100 |  |  |
| *Boloria selene* | 0 | 0 | 1 | 1 | 100 |  |  |
| *Chlosyne gorgone* | 0 | 0 | 1 | 1 | 100 |  |  |
| *Erynnis persius* | 0 | 0 | 1 | 1 | 100 |  |  |
| *Speyeria aphrodite* | 0 | 1 | 0 | 1 | 0 |  |  |
| richness | 17 | 20 | 26 | 28 |  |  |  |
| #/ year | 2646 | 4722 | 6415 | 13783 | 59.0 |  |  |

**Table** **S2. Estimated butterfly densities by grazer and year.**

We input average floral abundance and diversity for each grazer treatment and year into the most competitive model for each species to calculate a butterfly density estimate under those flower conditions. Floral variables are presented as standardized and unstandardized to allow comparison with density graphs. Density estimates are butterflies per hectare.

| Butterfly | Year | Grazer | Avg. floral abundance per transect (equivalent standardized value) | Estimated butterfly density/ha (lower and upper) |
| --- | --- | --- | --- | --- |
| *Colias eurytheme* | 2017 | Cattle | 562.64 (-0.13) | 43 (21 – 89) |
|  |  | Sheep | 84.56 (-0.6) | 42 (20 - 85) |
|  | 2018 | Cattle | 1140.81 (0.44) | 129 (72 – 233) |
|  |  | Sheep | 62.14 (-0.62) | 91 (48 – 171) |
|  | 2019 | Cattle | 2131 (1.41) | 104 (56 – 195) |
|  |  | Sheep | 201.22 (-0.49) | 38 (18 – 78) |
| *Plebejus melissa* | 2017 | Cattle | 562.64 (-0.13) | 208 (183 – 236) |
|  |  | Sheep | 84.56 (-0.6) | 142 (123 – 164) |
|  | 2018 | Cattle | 1140.81 (0.44) | 554 (510 – 602) |
|  |  | Sheep | 62.14 (-0.62) | 126 (109 – 146) |
|  | 2019 | Cattle | 2131 (1.41) | 75 (62 – 91) |
|  |  | Sheep | 201.22 (-0.49) | 22 (16 – 30) |
| *Pontia protodice* | 2017 | Cattle | 562.64 (-0.13) | 10 (6 -17) |
|  |  | Sheep | 84.56 (-0.6) | 5 (3 – 9) |
|  | 2018 | Cattle | 1140.81 (0.44) | 19 (13 – 28) |
|  |  | Sheep | 62.14 (-0.62) | 16 (10 – 24) |
|  | 2019 | Cattle | 2131 (1.41) | 73 (54 – 99) |
|  |  | Sheep | 201.22 (-0.49) | 39 (28 – 54) |
| *Vanessa cardui* | 2017 | Cattle | 562.64 (-0.13) | 28 (19 – 42) |
|  |  | Sheep | 84.56 (-0.6) | 23 (15 – 35) |
|  | 2018 | Cattle | 1140.81 (0.44) | 0 (0 – 0) |
|  |  | Sheep | 62.14 (-0.62) | 0 (0 – 10) |
|  | 2019 | Cattle | 2131 (1.41) | 137 (103 – 182) |
|  |  | Sheep | 201.22 (-0.49) | 139 (101 – 190) |
| Butterfly | Year | Grazer | Avg. floral diversity  per transect | Estimated butterfly density/ha (lower and upper) |
| *Colias philodice* | 2017 | Cattle | 0.27 | 75 (69 – 80) |
|  |  | Sheep | 0.22 | 77 (72 – 83) |
|  | 2018 | Cattle | 0.38 | 129 (121 – 138) |
|  |  | Sheep | 0.41 | 127 (119 – 135) |
|  | 2019 | Cattle | 0.48 | 253 (244 – 263) |
|  |  | Sheep | 0.44 | 249 (239 – 258) |


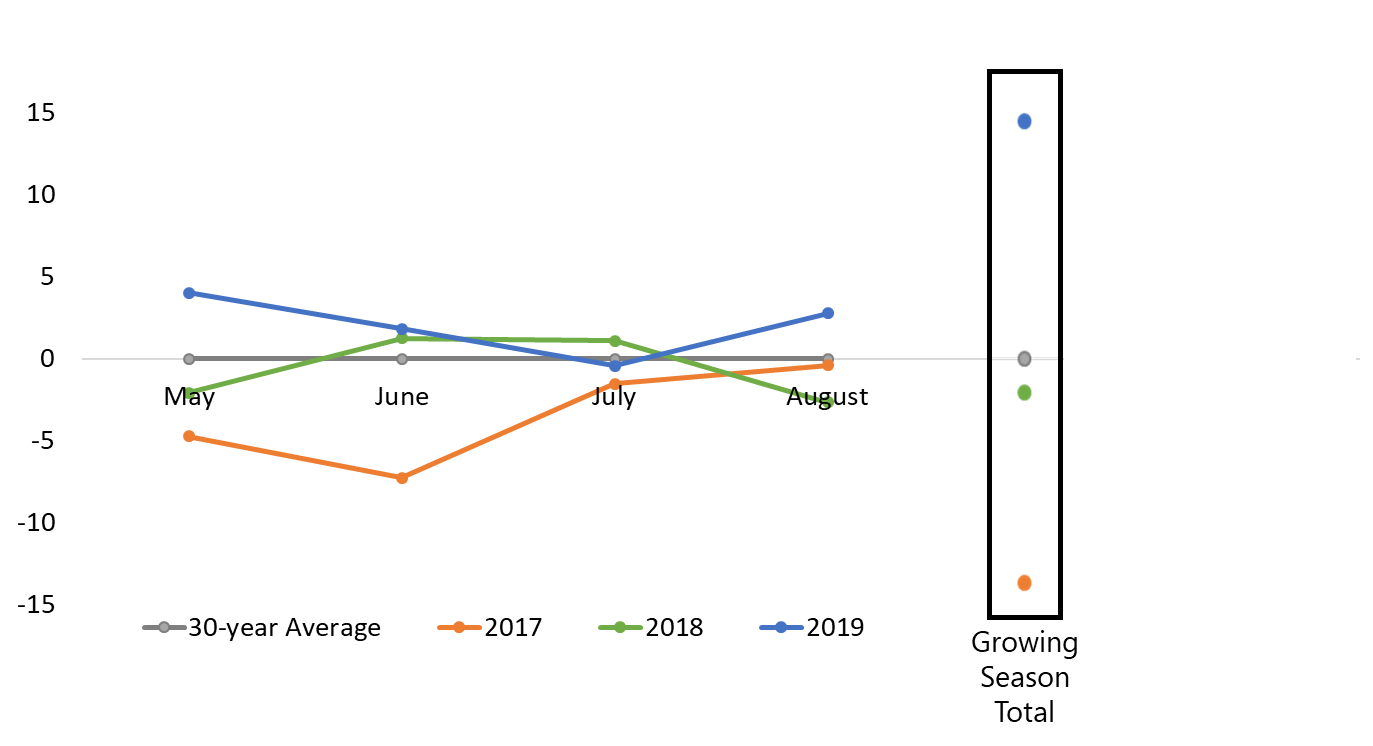


Figure S1. Precipitation during study period 2017-2019 compared to 30-year average.

In 2017, May – August precipitation was 13.6 cm below average. In 2018, it was 2 cm below average, and in 2019, it was 14.5 cm above average.


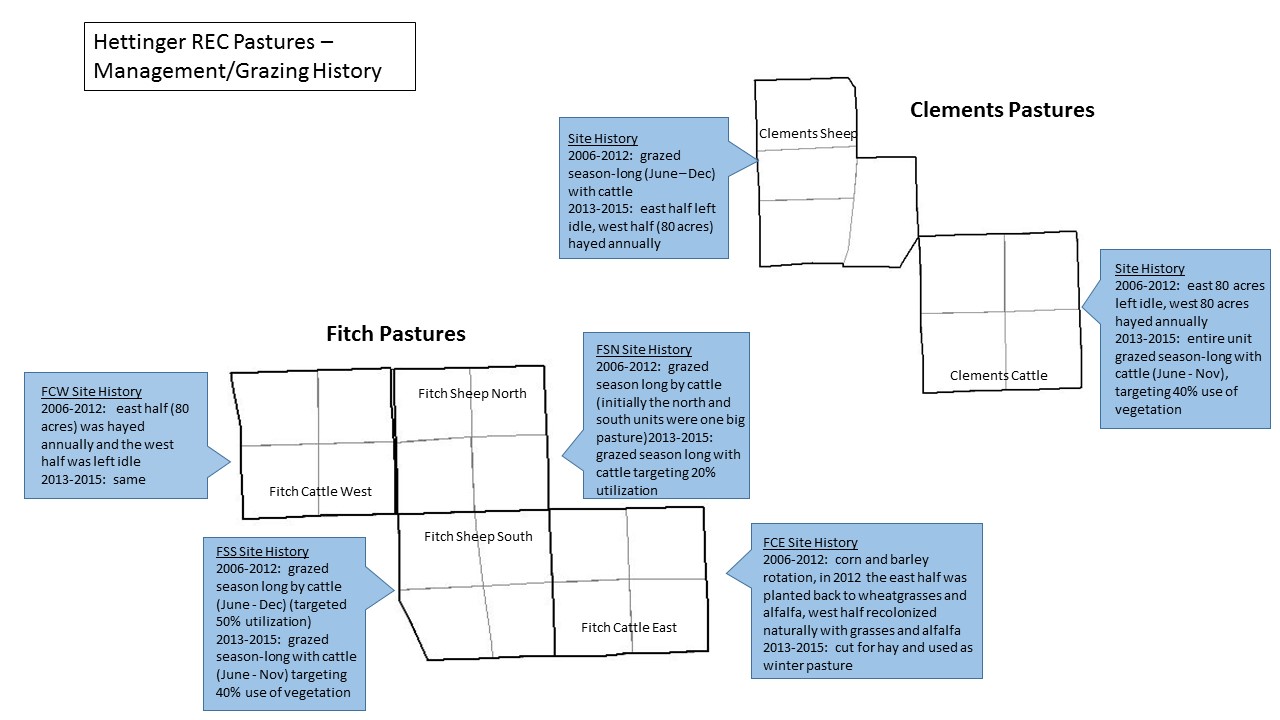


**Figure S2. Management history of study pastures (2006 – 2015).**


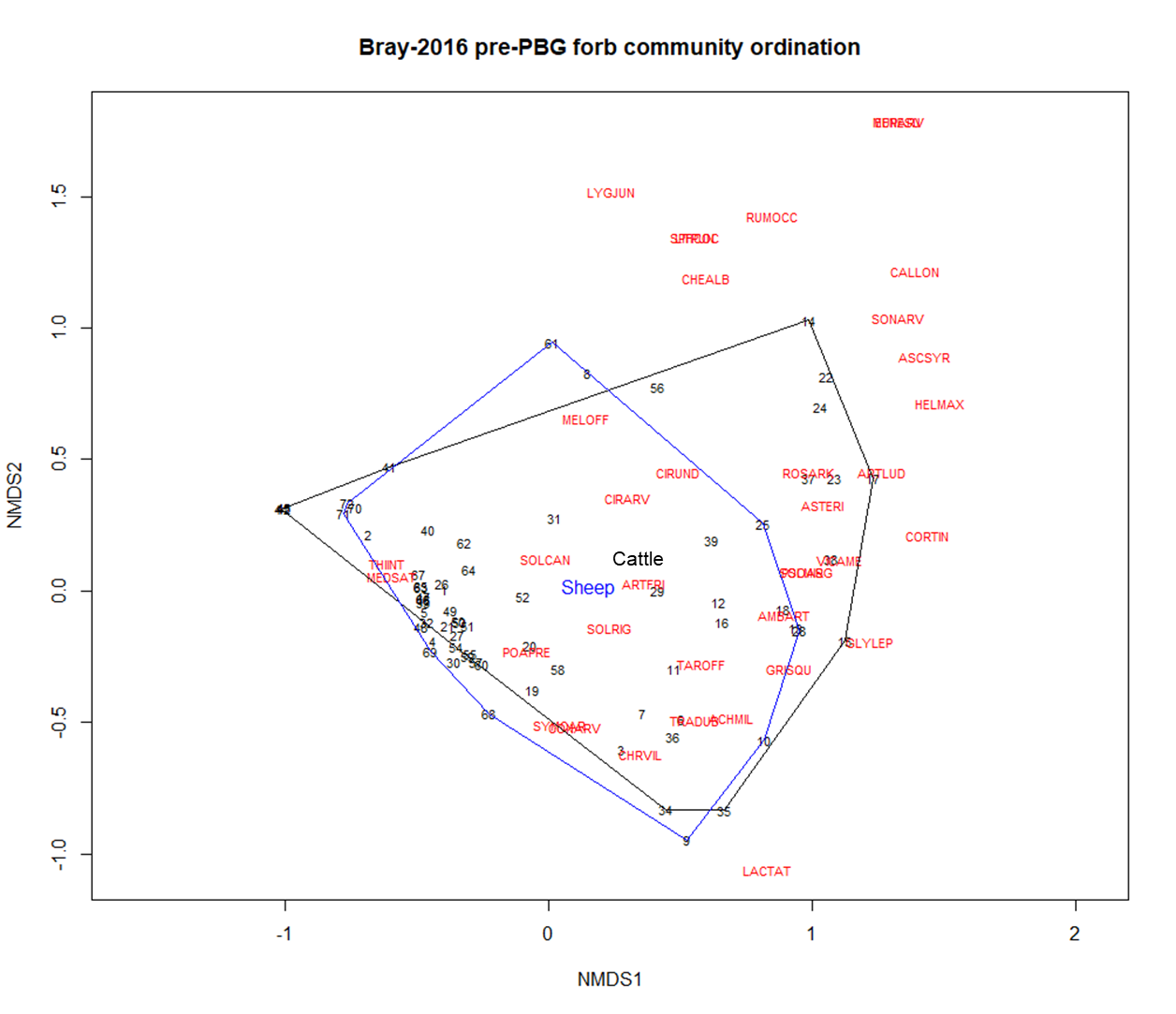


**Figure S3. NMDS Ordination of Forb Community in 2016.** Ordination has 3 axes (k=3) and a stress value of 0.1339). We used a Bray-Curtis dissimilarity index. PERMANOVA showed that there was no significant association between patterns in the forb community and whether a pasture was assigned to sheep or cattle treatment (*p* = 0.056).


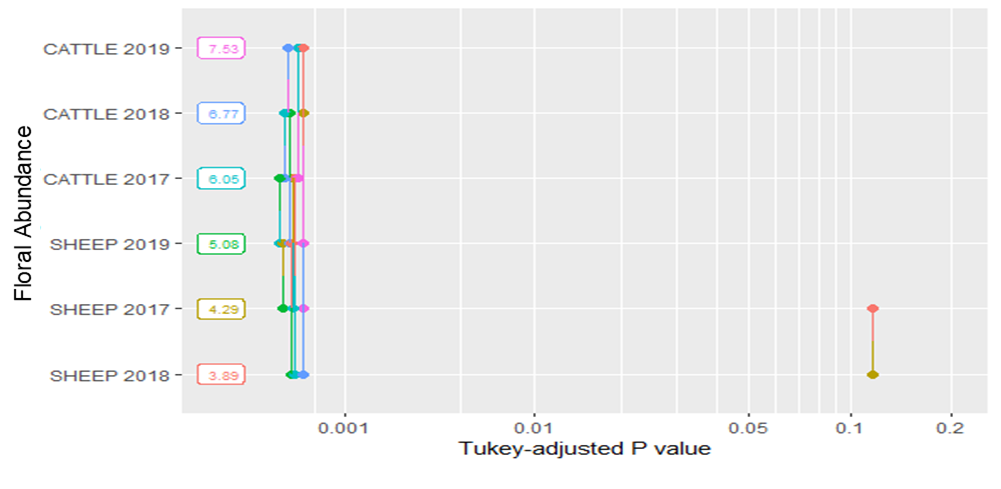
Figure S4. Pairwise Comparison of Grazer-Year Combinations for Floral Abundance 2017, 2018, 2019 at Hettinger Research Extension Center. Only floral abundances in 2017 sheep pastures and 2018 sheep pastures were not significantly different (*p* ≥ 0.05).


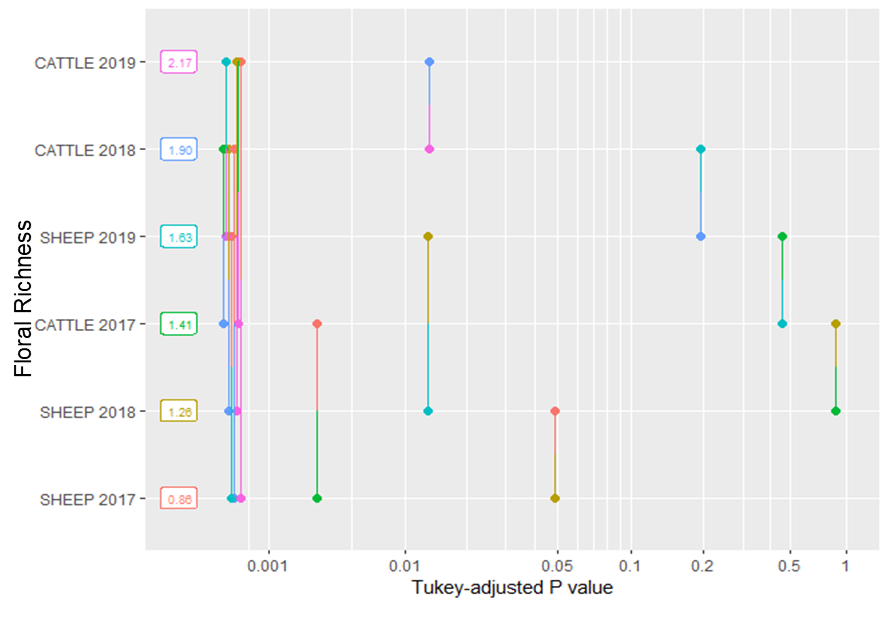


Figure S5. Pairwise Comparison of Grazer-Year Combinations for Floral Richness 2017, 2018, 2019 at Hettinger Research Extension Center. Floral richness was significantly higher each year within grazer treatments for both cattle and sheep.


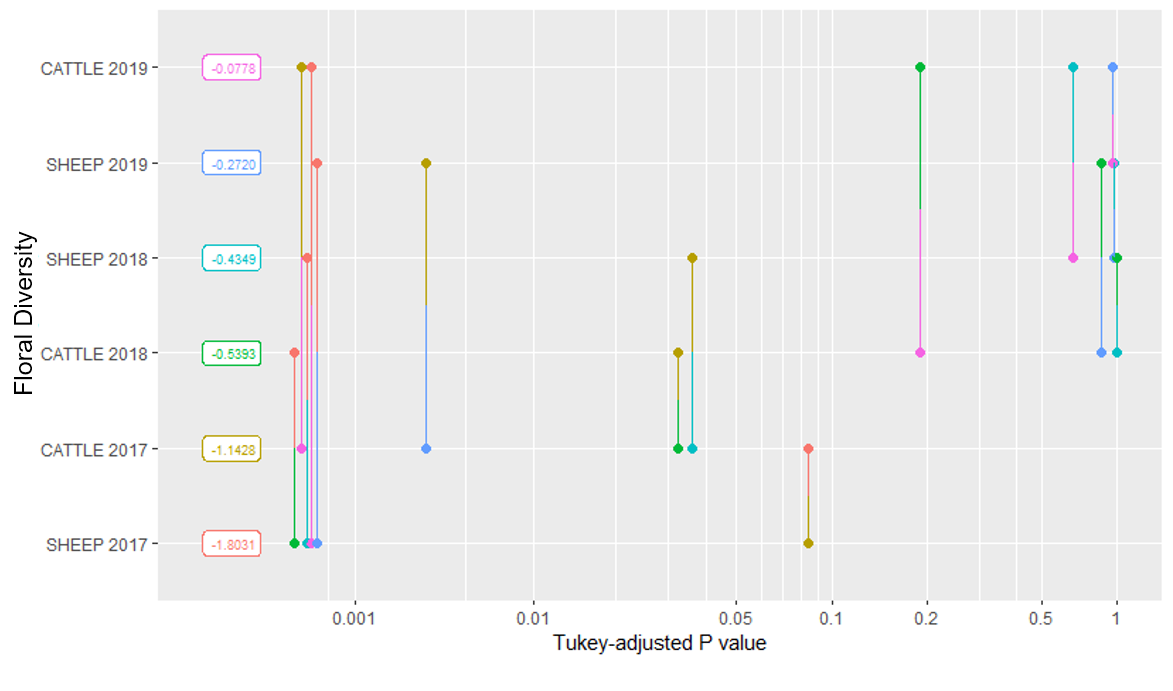


Figure S6. Pairwise Comparison of Grazer-Year Combinations for Floral Diversity 2017, 2018, 2019 at Hettinger Research Extension Center. In 2017, cattle pastures had significantly more floral diversity than sheep pastures (*p* = 0.0497)


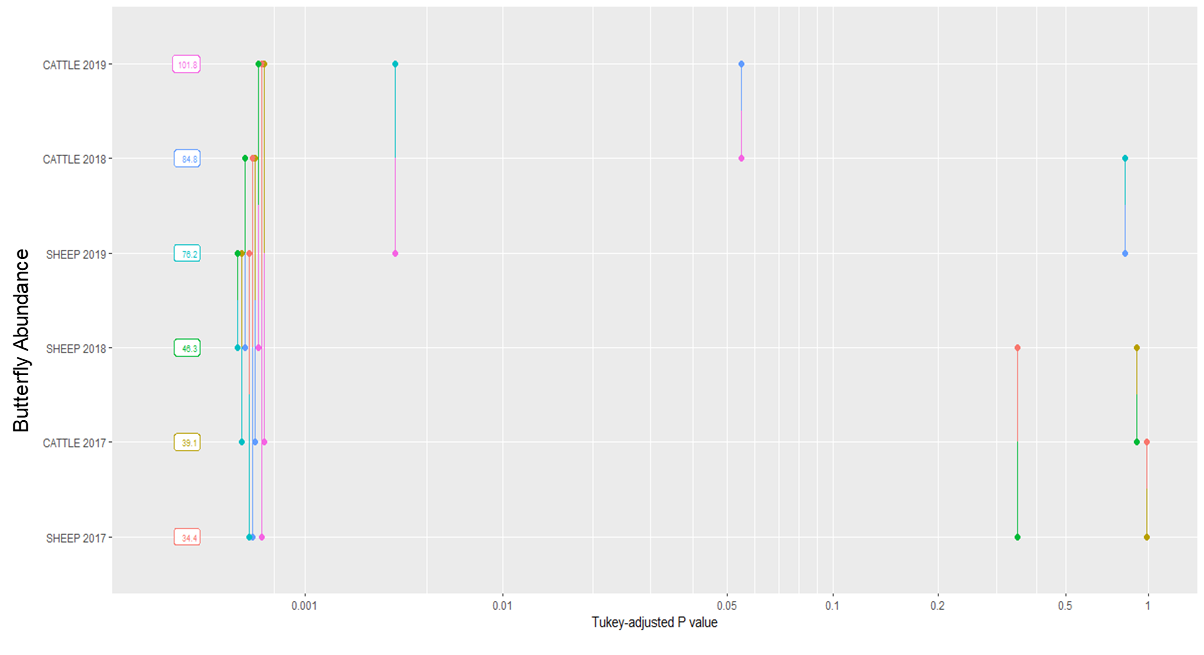


Figure S7. Pairwise Comparison of Grazer-Year Combinations for Butterfly Abundance 2017, 2018, 2019 at Hettinger Research Extension Center.


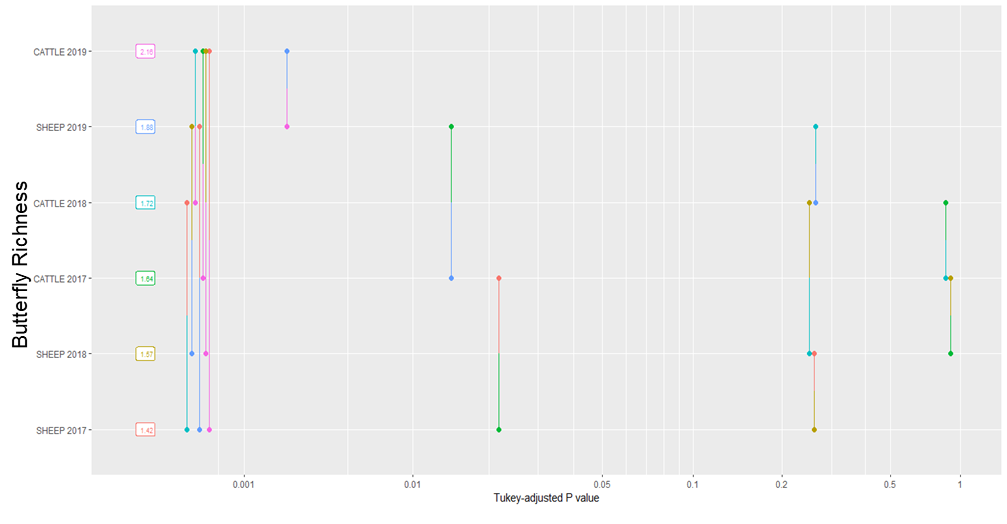
Figure S8. Pairwise Comparison of Grazer-Year Combinations for Butterfly Richness 2017, 2018, 2019 at Hettinger Research Extension Center.


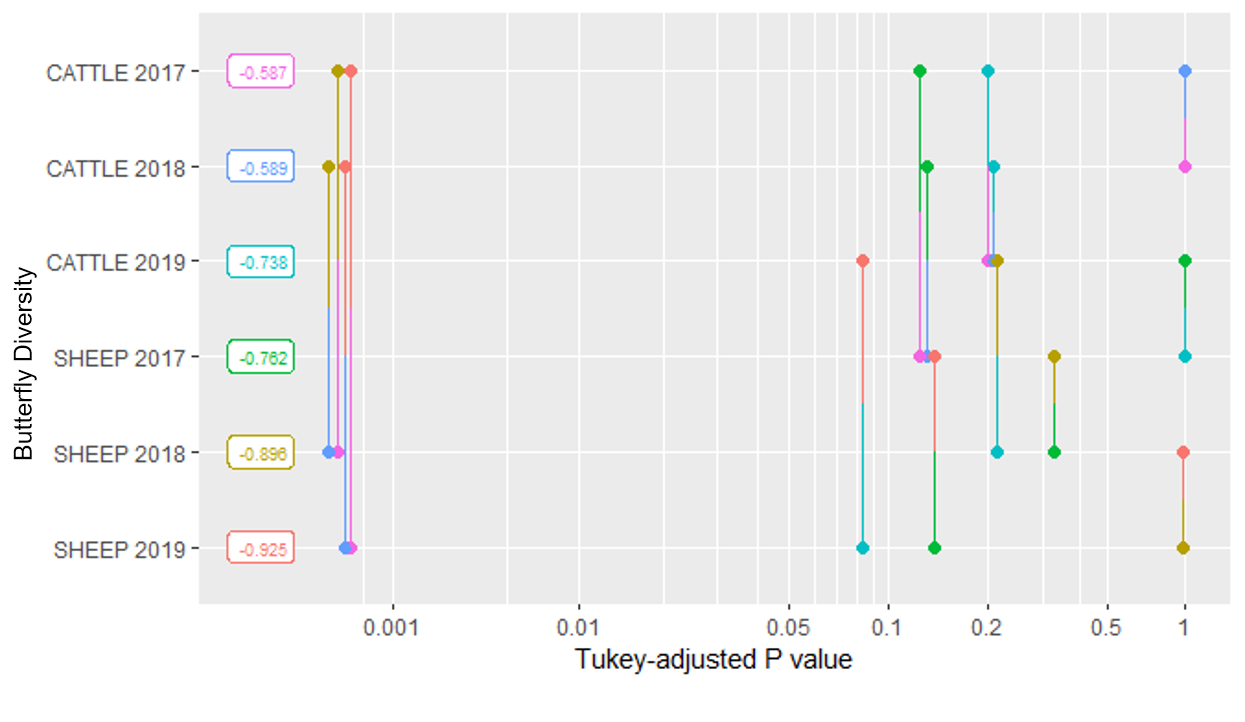


Figure S9. Pairwise Comparison of Grazer-Year Combinations for Butterfly Diversity 2017, 2018, 2019 at Hettinger Research Extension Center.

**
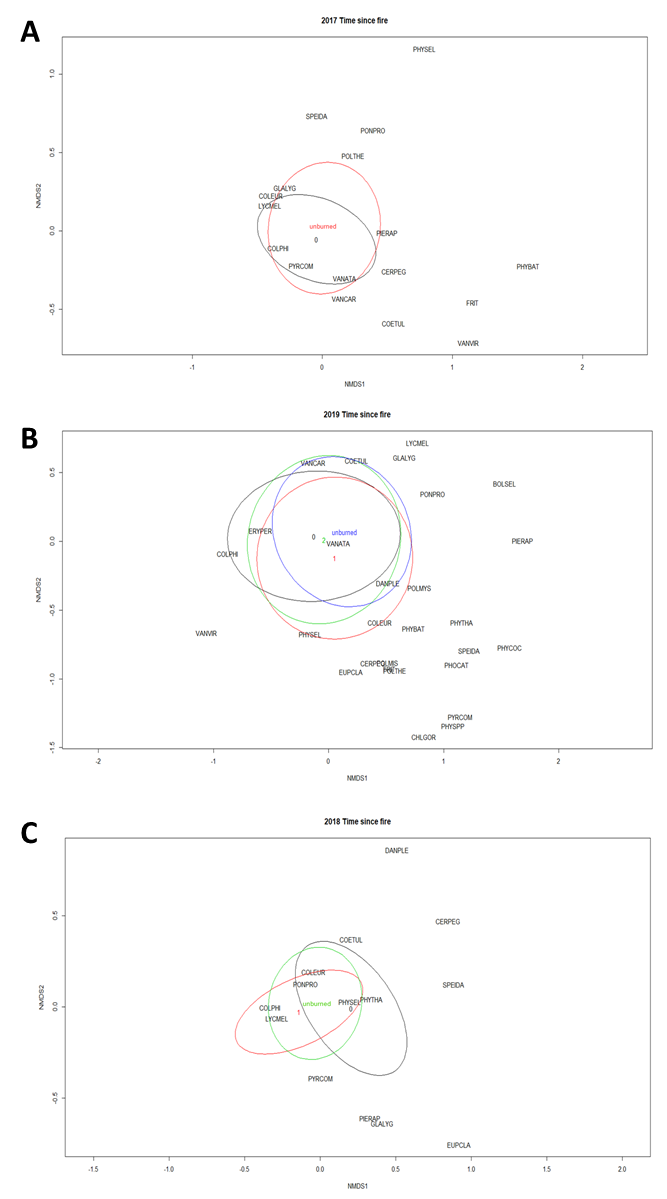
**

**Figure** **S10. Butterfly community NMDS ordination showing minimal variation in groupings by time since fire for 2017, 2018, 2019 at Hettinger Research Extension Center.**

Panel A shows a NMDS ordination using Bray-Curtis dissimilarity metric for the butterfly community (17 species) in 2017 (k = 4, stress = 0.14) and overlap between unburned, recently burned (0). Panel B shows a NMDS ordination using Bray-Curtis dissimilarity metric for the butterfly community (17 species) in 2018 (k = 4, stress = 0.13) and overlap between unburned, recently burned (0), and 1 year since fire (1). Panel C shows a NMDS ordination using Canberra dissimilarity metric for the butterfly community (25 species) in 2019 (k = 4, stress = 0.12) and overlap between unburned, recently burned (0), 1 year since fire (1), and 2 years since fire (2)
